# Autophagy–Ferroptosis Crosstalk in Dry Eye Disease: Context-Dependent Regulation of Ocular Surface Homeostasis

**DOI:** 10.3390/cells15171542

**Published:** 2026-08-26

**Authors:** Yuan Zhong, Jun Peng, Qinghua Peng

**Affiliations:** Faculty of Traditional Chinese Medicine, Hunan University of Chinese Medicine, Changsha 410208, China; zhongyuan@stu.hnucm.edu.cn (Y.Z.); pzj5600599@hnucm.edu.cn (J.P.)

**Keywords:** dry eye disease, ocular surface, autophagy, ferroptosis, autophagic flux, ferritinophagy, lipophagy, mitophagy, oxidative stress

## Abstract

**Highlights:**

**What are the main findings?**

- Autophagy is not uniformly protective in dry eye disease (DED): selective cargo turnover and lysosomal flux integrity shift the balance between ferroptotic pressure and cellular defense.
- Studies combining autophagy-related pathway perturbation with ferroptosis-related outcomes are concentrated in corneal and conjunctival epithelial models, whereas evidence from lacrimal and meibomian tissues remains less direct.

**What are the implications of the main findings?**

- Static autophagy markers are insufficient; mechanistic studies should integrate flux measurements with iron-dependent phospholipid peroxidation and ferroptosis-selective rescue.
- Therapeutic strategies should target defined pathways and ocular compartments, because global activation or inhibition of autophagy may produce opposing effects on ferroptotic injury.

**Abstract:**

Dry eye disease (DED) exposes the ocular surface to persistent hyperosmolar, oxidative, metabolic, and inflammatory stress that may increase susceptibility to ferroptosis. Whether autophagy promotes or limits ferroptotic injury in DED remains unresolved. This review critically evaluates evidence from corneal and conjunctival epithelia, lacrimal glands, and meibomian glands, using non-ocular models only to interpret mechanisms not yet resolved in ocular tissues. We propose a threshold-balance framework in which autophagy has context-dependent effects. Ferritinophagy, lipophagy, and impaired autophagic flux can expand the labile iron pool, mobilize peroxidizable lipids, and promote lipid peroxidation. Conversely, intact autophagic–lysosomal flux and mitophagy can remove damaged cargo, limit mitochondrial oxidative stress, and preserve membrane antioxidant defenses. The balance between these processes may determine whether stressed ocular-surface cells adapt or progress toward ferroptotic injury, with consequences for epithelial barrier integrity, tear-film homeostasis, glandular secretion, and inflammation. Studies combining autophagy-related pathway perturbation with ferroptosis-related outcomes are concentrated in ocular-surface epithelial models, whereas this relationship has been less directly examined in lacrimal and meibomian tissues. Establishing causality will require flux-aware measurements, targeted pathway perturbation, tissue-resolved models, and functional validation in human samples. Such studies should clarify when and where autophagy–ferroptosis crosstalk is therapeutically actionable in DED.

## 1. Introduction

Dry eye disease (DED) is a multifactorial, symptomatic disease characterized by a loss of homeostasis of the tear film and/or ocular surface. Tear-film instability and hyperosmolarity, ocular-surface inflammation and damage, and neurosensory abnormalities are major etiological contributors [1,2]. This definition has shifted the field beyond a simple tear-deficiency model. DED is now viewed as a disorder of the broader lacrimal functional unit, which includes the tear film, ocular-surface epithelia, lacrimal and meibomian glands, immune cells, and sensory nerves. The cellular processes that determine whether stressed ocular-surface cells adapt, recover, or undergo regulated cell death remain incompletely understood.

The ocular surface is continuously exposed to environmental and metabolic stress. Evaporation, tear hyperosmolarity, impaired tear clearance, and other external insults can increase reactive oxygen species (ROS) production and disrupt epithelial metabolism. Early para-inflammatory and stress-adaptive responses may help preserve barrier function. With persistent or severe stress, antioxidant and degradative capacity may become insufficient, amplifying inflammation and lowering the threshold for cell death [3,4]. A mechanistic account of DED must explain both how stress arises and how cellular compensation gives way to tissue injury.

Ferroptosis may provide one mechanism underlying this transition. It is a form of regulated cell death driven by iron-dependent membrane phospholipid peroxidation when cellular antioxidant defenses fail to restrain lipid peroxide accumulation [5,6]. Several features of the ocular surface in DED are compatible with a ferroptosis-permissive state, including ROS accumulation and mitochondrial dysfunction, disturbed iron homeostasis, impairment of the solute carrier family 7 member 11 (SLC7A11)–glutathione (GSH)–glutathione peroxidase 4 (GPX4) antioxidant axis, and increased availability of peroxidizable lipid substrates [7,8,9,10,11]. Experimental studies have reported ferroptosis-related changes in corneal epithelial cells, conjunctival epithelial cells, and lacrimal glands under DED-associated stress [8,9,10,11]. The criteria used to identify ferroptosis and the extent to which causal relationships have been tested vary among models.

Autophagy is a lysosome-dependent quality-control pathway that degrades and recycles damaged proteins, organelles, lipid droplets, and other cytoplasmic cargo. It supports epithelial homeostasis, mitochondrial quality control, innate immune regulation, and adaptation to metabolic stress [12,13,14]. Autophagy can also modify ferroptotic susceptibility through distinct and potentially opposing processes [12]. Nuclear receptor coactivator 4 (NCOA4)-dependent ferritinophagy releases stored iron, whereas lipophagy mobilizes oxidizable lipid substrates [8,11]. When autophagic flux is impaired, sequestosome 1 (SQSTM1; also known as p62) can accumulate and promote acyl-CoA synthetase long-chain family member 4 (ACSL4)-dependent lipid remodeling [15]. By contrast, intact autophagic–lysosomal flux and mitophagy may preserve cellular quality control by removing damaged cargo and ROS-producing mitochondria [12,13,14]. Direct evidence that these protective functions restrain ferroptosis in DED is still limited.

The central issue is therefore not whether autophagy is globally increased or decreased in DED but which autophagic process modifies ferroptotic susceptibility in a specific ocular cell type and disease context. Ferritin and lipid-droplet turnover may increase the availability of iron and lipid substrates, whereas intact autophagic–lysosomal flux, mitophagy, and antioxidant systems may limit cellular damage. Previous reviews have examined autophagy in DED, regulated cell death at the ocular surface, ferroptosis across ophthalmic diseases, organelle involvement in DED-associated ferroptosis, and ferroptosis-targeted therapeutic opportunities [13,16,17,18,19]. The present review focuses more specifically on the mechanistic links between autophagy and ferroptosis and organizes the available evidence within a threshold-balance framework.

This focused narrative review examines evidence from DED models and from corneal, conjunctival, lacrimal, and meibomian tissues. Evidence from non-ocular and related exocrine tissues is used only to interpret mechanisms that remain unresolved at the ocular surface and is explicitly identified as contextual support. In interpreting the selected literature, we distinguish direct mechanistic evidence, associative or one-directional evidence, single-process evidence, and contextual evidence according to the experimental relationship evaluated. These categories describe experimental scope and do not constitute a formal evidence-grading system.

### Literature Search and Evidence Selection

A structured PubMed search was used to support this focused narrative review. The literature search was initially completed on 15 July 2026. An updated search using five complementary search blocks was performed on 22 August 2026. The search covered PubMed from database inception to the update date. No date, language, or publication-type filters were applied. The search blocks combined DED and relevant ocular compartments with autophagy, autophagic flux, lysosomal function, ferritinophagy, lipophagy, mitophagy, ferroptosis, iron metabolism, phospholipid peroxidation, and related antioxidant-defense pathways. The complete search strings and the results of each search block are provided in Supplementary Table S1.

The five search blocks returned 470 records before deduplication. After the results were combined by PMID, 359 unique records remained and were screened by title and abstract. Records were retained for detailed relevance assessment when they examined DED or a relevant ocular compartment together with an autophagy-, lysosome-, selective-autophagy-, ferroptosis-, iron-, or phospholipid-peroxidation-related outcome. Non-ocular and related exocrine studies were considered only when they addressed a defined mechanism needed to interpret an unresolved ocular-surface question.

Records were excluded during title and abstract screening when they concerned an unrelated disease or anatomical compartment, lacked a sufficiently specific mechanistic outcome, mentioned a relevant pathway only incidentally, or evaluated an intervention without relevant pathway assessment. Records that did not contribute directly to the focused interpretation were also excluded. When relevance could not be determined reliably from the title and abstract, the record was retained for detailed assessment. On this basis, 247 records were excluded, and 112 records underwent detailed relevance assessment.

During detailed assessment, records were selected for the focused synthesis when they provided DED-related experimental evidence concerning autophagy, lysosomal function, selective autophagy, or ferroptosis; examined an autophagy-related perturbation together with ferroptosis-related outcomes; or provided mechanistic evidence needed to interpret a specific unresolved ocular pathway. Sixty-eight records were not used in the final synthesis because they addressed related systemic or exocrine models without a necessary contribution to the ocular-surface interpretation, focused on other ocular diseases, provided only predictive or marker-level evidence, or were less directly informative than the studies retained for the same mechanistic point. Forty-four records identified through the structured PubMed search were selected for the focused synthesis.

An additional 38 references were identified through targeted reference checking of key studies and relevant reviews. These references were used for disease definitions, foundational mechanisms, methodological guidance, and complementary experimental or clinical context. They were not counted among the 44 records selected directly from the structured PubMed search. The revised manuscript therefore contains 82 references in total. Because PubMed was the only bibliographic database searched, studies indexed exclusively elsewhere may have been missed. This search was intended to improve the transparency of literature selection for a focused narrative synthesis. It was not designed as a systematic review or a formal quantitative evidence-grading process. Accordingly, the experimental relationships summarized in this review are interpreted qualitatively rather than as a formal evidence-grading system.

## 2. Ferroptosis and Autophagy at the Ocular Surface in Dry Eye Disease

Ocular-surface homeostasis is maintained by an integrated lacrimal functional unit comprising the tear film, corneal and conjunctival epithelia, lacrimal and meibomian glands, together with resident immune cells and interconnected sensory nerves [1,2,4]. In DED, tear-film instability and hyperosmolarity repeatedly expose these compartments to oxidative, metabolic, and inflammatory stress [20,21]. Evaporation and impaired tear clearance can further amplify tear hyperosmolarity, while airborne pollutants promote ocular-surface oxidative, inflammatory, and mitochondrial injury [20,21,22,23,24]. Experimental cigarette-smoke and blue-light models additionally demonstrate disturbed iron handling and increased lipid peroxidation [8,25]. Although these changes do not by themselves establish ferroptosis, they create conditions compatible with iron-dependent phospholipid peroxidation [5,6].

**Figure 1:**
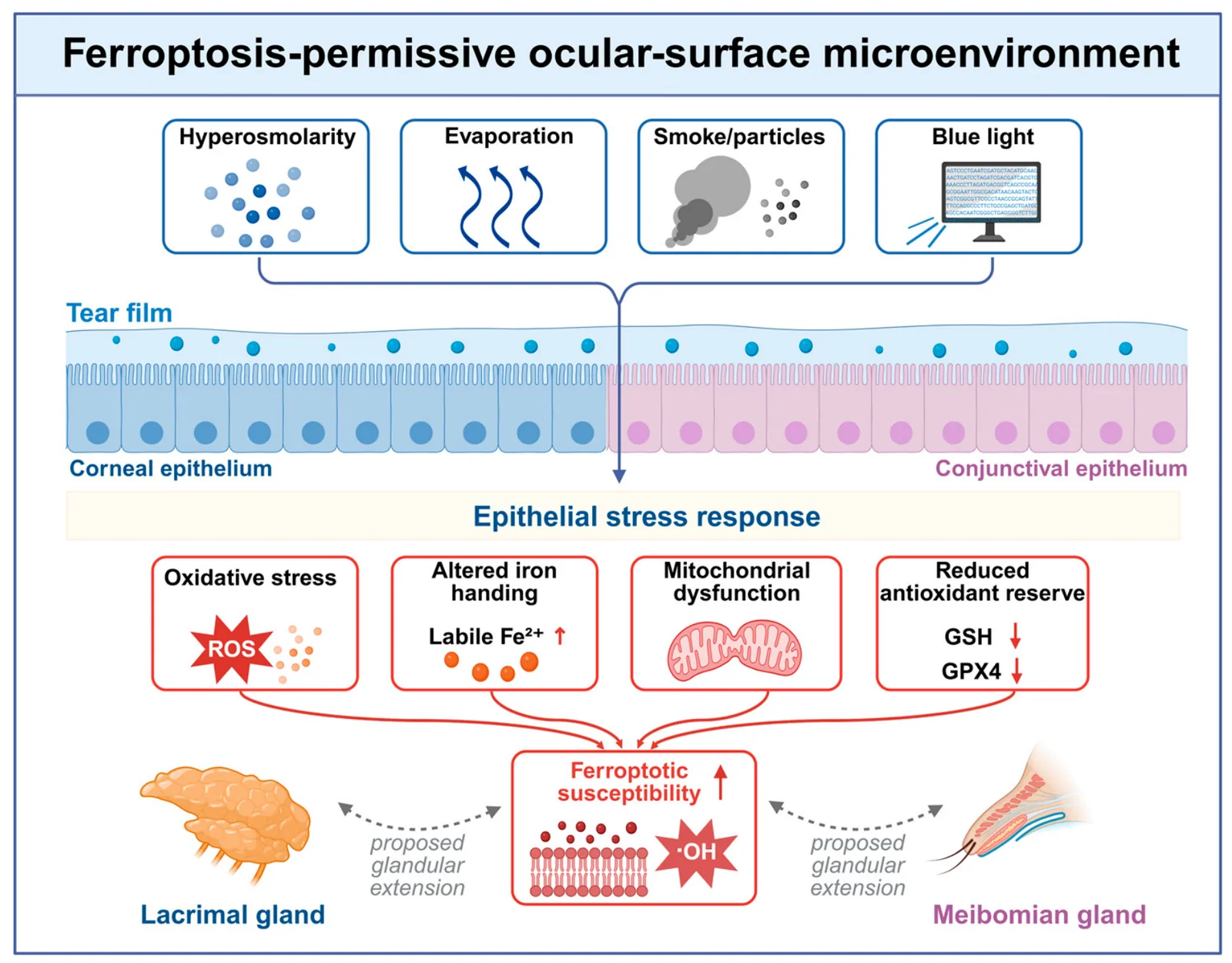
Ferroptosis-permissive microenvironment at the ocular surface in dry eye disease. Tear-film hyperosmolarity, evaporation, and exposure to smoke, airborne particles, or blue light impose oxidative and metabolic stress on the corneal and conjunctival epithelia. In this figure, “smoke/particles” encompasses cigarette smoke and airborne particulate matter. These stressors may increase ROS production, disrupt iron homeostasis and mitochondrial function, and reduce glutathione-dependent antioxidant capacity. Together, these changes create a microenvironment that increases susceptibility to iron-dependent phospholipid peroxidation and ferroptotic injury. Lacrimal- and meibomian-gland involvement is presented as a proposed tissue-level extension because the complete autophagy–ferroptosis chain has not been established in these compartments. Solid arrows indicate relationships synthesized from the available evidence, whereas dashed arrows indicate proposed extensions requiring further validation. •OH, hydroxyl radical; Fe^2+^, ferrous iron; GPX4, glutathione peroxidase 4; GSH, glutathione; ROS, reactive oxygen species.

The same microenvironment places sustained demand on autophagic quality control. Ocular epithelial and secretory cells rely on lysosomal degradation to remove damaged proteins, organelles, and lipid stores and to adapt to nutrient and redox stress [12,13,14]. Autophagy is not uniformly protective: selective degradation of ferritin or lipid droplets may increase the availability of iron or lipid substrates for ferroptosis, and the overall effect depends on both cargo identity and completion of lysosomal degradation. Figure 1 summarizes the stressors and tissue-level mechanisms that may render the ocular surface in DED susceptible to ferroptotic injury.

### 2.1. Ferroptotic Susceptibility in DED

Ferroptosis requires the convergence of redox-active iron, oxidizable membrane phospholipids, and insufficient detoxification of phospholipid hydroperoxides [5,6]. GPX4 detoxifies phospholipid hydroperoxides, whereas ACSL4 increases ferroptotic susceptibility by promoting the incorporation of polyunsaturated fatty acids into membrane phospholipids [26,27]. Ferroptosis suppressor protein 1 (FSP1) provides a parallel defense by reducing ubiquinone, also known as coenzyme Q, to ubiquinol, a lipophilic radical-trapping antioxidant [28,29].

Because no single molecular marker establishes ferroptosis, increases in ROS, malondialdehyde, 4-hydroxynonenal, or ACSL4, together with reduced GPX4, are best described as ferroptosis-associated. A stronger mechanistic assignment requires evidence of iron dependence, phospholipid peroxidation, and rescue by ferroptosis-selective pharmacological agents or appropriate genetic perturbation [5,6]. Whenever possible, these measurements should be linked to cell injury and an ocular functional outcome.

DED-relevant studies provide evidence from several ocular compartments. Hyperosmolar or desiccating-stress models demonstrate altered iron handling and increased lipid peroxidation in corneal epithelial cells [10,11,15,30,31,32]. Other DED studies demonstrate impairment or therapeutic restoration of SLC7A11–GSH–GPX4-related defense [10,32,33,34]. In one hyperosmolar corneal epithelial model, ferrostatin-1, deferoxamine, and astaxanthin suppressed ferroptosis-related injury, whereas astaxanthin restored autophagic flux measured using a tandem fluorescent LC3 reporter [34]. Because autophagy was not independently perturbed, this study supports an associative or one-directional relationship from ferroptosis inhibition to flux recovery rather than direct causal autophagy–ferroptosis crosstalk. Cigarette-smoke exposure promotes ferritin cleavage and iron accumulation, and Yes-associated protein (YAP)–ACSL4 signaling has been implicated in smoke-associated corneal epithelial ferroptosis [25,35]. Blue-light exposure induces NCOA4-dependent ferritinophagy and iron overload in conjunctival epithelial cells [8]. Corneal denervation is accompanied by ferroptosis-associated lacrimal acinar injury and reduced tear secretion [9]. Oxidative stress is also implicated broadly in lacrimal-gland and ocular-surface injury [36]. Ferroptosis in meibomian-gland progenitor cells contributes to experimental meibomian gland dysfunction [37].

A more specific mechanistic interpretation is possible when molecular changes are linked to iron- and lipid-peroxidation-dependent injury, ferroptosis-selective rescue, and an ocular functional endpoint. Several studies include some of these experimental features, although the combinations of measurements and interventions vary among models. Blue-light and cigarette-smoke paradigms represent specific environmental injuries and cannot be generalized to all DED phenotypes. Studies in lacrimal and meibomian tissues support ferroptotic involvement in secretory compartments, but the autophagy component has not been tested directly in these models. These distinctions are retained throughout this review.

Ferroptotic susceptibility may also vary across DED phenotypes and experimental models. Tear hyperosmolarity increases mitochondrial and inflammatory stress, whereas aqueous-deficient and evaporative phenotypes place different demands on lacrimal and meibomian compartments. Multi-omics studies collectively implicate interconnected oxidative-stress, lipid-metabolic, immune-inflammatory, and tissue-repair pathways in DED [38]. Pathway enrichment alone cannot establish active ferroptosis; candidate signatures require validation using labile-iron measurements, oxidized phospholipid species, ferroptosis-selective rescue, and functional ocular-surface outcomes.

### 2.2. Autophagy and Autophagic Flux in DED

**Figure 2:**
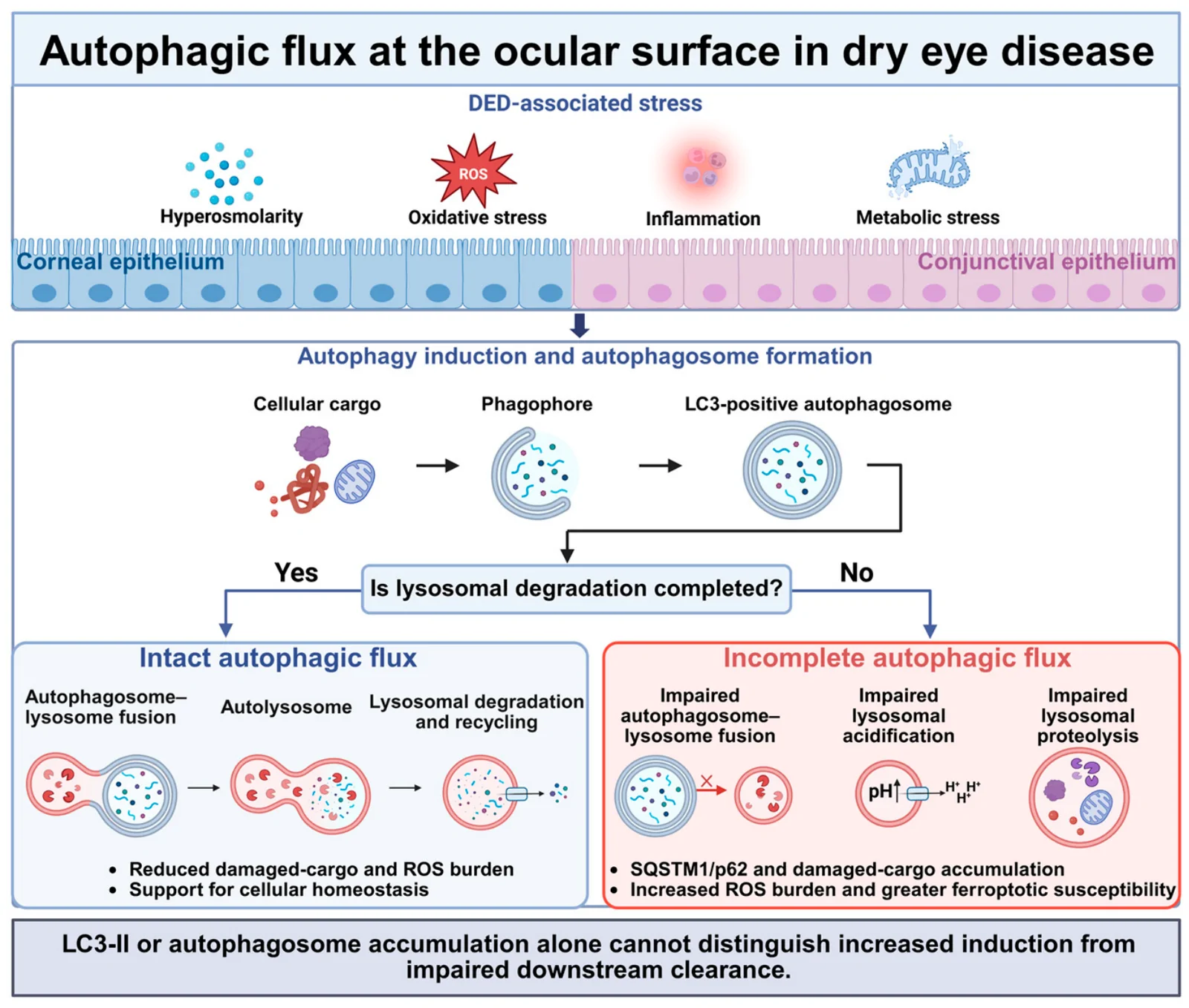
Autophagic flux at the ocular surface in dry eye disease. Hyperosmolar, oxidative, inflammatory, and metabolic stress can affect autophagy induction, autophagosome formation, autophagosome–lysosome fusion, lysosomal acidification, cargo degradation, and recycling. With intact autophagic flux, damaged proteins and organelles are delivered to lysosomes for degradation, thereby reducing the ROS burden and supporting cellular homeostasis. When fusion, acidification, or proteolysis is impaired, autophagosomes and damaged cargo accumulate. Incomplete flux may consequently promote SQSTM1/p62 accumulation, oxidative stress, and greater ferroptotic susceptibility. Increased LC3-II abundance or autophagosome accumulation alone cannot distinguish enhanced autophagy induction from impaired downstream clearance. Black arrows indicate pathway progression. Blue and red arrows and outlines denote intact and incomplete autophagic flux, respectively. The red cross indicates impaired autophagosome–lysosome fusion, whereas pH↑ indicates impaired lysosomal acidification. DED, dry eye disease; LC3, microtubule-associated protein 1 light chain 3; LC3-II, lipidated form of LC3; ROS, reactive oxygen species; SQSTM1/p62, sequestosome 1.

Macroautophagy is a dynamic degradative pathway that maintains cellular homeostasis by removing damaged proteins, organelles, and other cytoplasmic cargo. It involves initiation, phagophore formation, generation of microtubule-associated protein 1 light chain 3 (LC3)-positive autophagosomes, autophagosome–lysosome fusion, lysosomal degradation, and recycling of cellular constituents [12,13,14]. Effective clearance depends on completion of this sequence rather than autophagosome formation alone. At the ocular surface, hyperosmolar, oxidative, inflammatory, and metabolic stress may disrupt different steps of the pathway. Productive autophagic flux can support adaptation by clearing damaged cargo, whereas failure to complete lysosomal degradation permits damaged proteins and organelles to accumulate (Figure 2).

A central interpretive challenge is that increased LC3-II abundance or autophagosome accumulation can represent two biologically distinct states. The first is enhanced autophagy induction with preserved lysosomal degradation. The second is impaired pathway completion, in which autophagosomes accumulate because autophagosome–lysosome fusion or lysosomal degradation is inefficient. Static LC3-II measurements therefore cannot distinguish increased autophagic activity from downstream blockade. SQSTM1/p62 accumulation may support impaired cargo clearance, but its abundance is also influenced by transcriptional regulation and should not be interpreted in isolation.

Flux-aware assessment should combine LC3-II turnover in the presence and absence of lysosomal inhibition with SQSTM1/p62 turnover and validated autophagic-flux reporters. When downstream blockade is suspected, lysosomal acidification and proteolytic competence should also be assessed [39]. Figure 2 summarizes this distinction and the principal approaches used to assess pathway completion. This methodological caution is important because terms such as “autophagy activation” and “autophagy inhibition” cannot be inferred reliably from static protein measurements alone.

Experimental studies generally associate preserved or restored autophagic function with improved ocular-surface homeostasis. Hyperosmolarity-induced oxidative stress can compromise autophagic function, particularly mitophagy, in corneal epithelial cells [40]. Flux-aware studies further indicate that hyperosmolar stress can affect downstream autophagic clearance in corneal epithelial cells. DNA damage-inducible transcript 4 (DDIT4) induction was associated with oxidative stress and impaired autophagic flux, whereas DDIT4 inhibition improved flux-related readouts and preserved epithelial-cell viability [41]. In a separate hyperosmolarity-based model, long noncoding RNA MIAT neighbor (MIATNB) knockdown impaired autophagic degradation, as assessed using tandem fluorescent LC3 reporting and pharmacological inhibition, and increased epithelial-cell apoptosis [42]. These studies support the relevance of flux completion to epithelial homeostasis in DED, but neither examined ferroptosis.

Several endogenous regulators and pharmacological interventions alleviate experimental DED phenotypes in association with autophagy-related or autophagy–lysosomal changes [43,44,45,46,47,48,49,50,51,52]. The extent of flux validation varies across these studies, and changes in LC3-II or SQSTM1/p62 alone do not demonstrate enhanced autophagic flux.

PTEN-induced kinase 1 (PINK1)-dependent mitophagy has been associated with reduced mitochondrial injury under hyperosmolar stress [7,47]. By contrast, S100 calcium-binding protein A9 (S100A9)–Toll-like receptor 4 (TLR4)-mediated autophagic blockade aggravates ocular-surface damage [52]. Together, these studies support a role for autophagic quality control in DED. Because most studies did not test ferroptosis dependence, improvements accompanied by autophagy-related marker changes support autophagy-associated protection rather than direct autophagy–ferroptosis crosstalk.

Autophagy also contributes to lacrimal-gland development, mitochondrial homeostasis, and secretory competence in organoid and acinar models [53,54]. These findings extend the relevance of autophagic quality control beyond ocular-surface epithelia but stop short of linking altered autophagy to ferroptosis in lacrimal or meibomian tissues.

Meaningful comparison across DED studies requires reporting stress duration, lysosomal-inhibitor conditions, reporter behavior, cargo turnover, and the tissue or cell compartment examined. Broad labels such as “autophagy activation” or “autophagy inhibition” are otherwise insufficient to predict ferroptotic susceptibility. We interpret a relationship as direct mechanistic evidence when genetic or pharmacological perturbation of a defined autophagy-related component alters ferroptosis-related injury, ideally together with evidence of iron-dependent phospholipid peroxidation and ferroptosis-selective rescue. Associative or one-directional evidence refers to studies that assess both processes in parallel, or perturb one process and observe a change in the other without reciprocal pathway testing. Single-process evidence refers to studies that examine autophagy, mitophagy, or ferroptosis alone within DED-relevant models, whereas contextual evidence refers to non-ocular or related exocrine studies used to interpret mechanisms that remain unresolved at the ocular surface. These categories describe the experimental relationships evaluated and do not constitute a formal evidence-grading system. Table 1 provides a qualitative map of autophagy-related findings, ferroptosis-related findings, and their experimentally investigated relationships in DED-relevant models. This framework provides the basis for the pro-ferroptotic and protective mechanisms discussed in Section 3 and Section 4.

## 3. Pro-Ferroptotic Consequences of Autophagic Dysregulation in DED

DED-focused studies have linked three autophagy-related pathways to increased ferroptotic injury. NCOA4-dependent ferritinophagy releases stored iron, ATG5-dependent lipophagy mobilizes lipid substrates, and impaired autophagic flux permits SQSTM1/p62 accumulation and ACSL4-associated lipid peroxidation [8,11,15]. The first two involve selective cargo degradation, whereas the third reflects incomplete autophagic flux.

### 3.1. NCOA4-Dependent Ferritinophagy Expands the Labile Iron Pool

NCOA4 binds ferritin heavy chain 1 (FTH1) and directs ferritin toward lysosomal degradation, thereby releasing stored iron [55,56,57]. In blue-light-associated DED models, NCOA4 inhibition or knockdown reduced iron accumulation, lipid peroxidation, conjunctival epithelial injury, and ocular-surface abnormalities [8]. These findings directly link NCOA4-dependent ferritin turnover to ferroptotic injury in this exposure-specific model.

The relevant variable is the labile, redox-active iron pool, particularly Fe^2+^, rather than total cellular iron. NCOA4 expression or ferritin loss alone cannot establish ferritinophagic flux. Ferritin turnover should be linked to lysosomal dependence, expansion of the labile iron pool, phospholipid peroxidation, and ferroptosis-selective rescue. Smoke-exposed corneal epithelial cells also demonstrate ferritin cleavage and Fe^2+^ accumulation [35], while YAP–ACSL4 signaling has been implicated in smoke-associated ferroptosis [25]. NCOA4 dependence has not been shown in smoke-exposure models. Ferritinophagy is established in experimental blue-light injury but remains a candidate mechanism in other iron-loading DED contexts.

Ferritinophagy operates alongside cellular iron uptake, export, and storage. Increased transferrin-receptor-mediated uptake or reduced ferroportin-mediated export can expand the same labile iron pool, whereas ferritin synthesis can buffer it. Studies attributing iron accumulation to NCOA4 should therefore evaluate whether altered iron uptake or export makes an independent contribution. This distinction also matters therapeutically. Although iron chelation can reduce redox-active iron, indiscriminate chelation could disrupt iron-dependent epithelial metabolism.

### 3.2. ATG5-Dependent Lipophagy Mobilizes Peroxidizable Lipid Substrates

Lipid droplets temporarily sequester fatty acids away from oxidizable membrane phospholipids. ATG5-dependent lipophagy mobilizes these stores and increases the fatty acid pool available for membrane remodeling. In DED-relevant corneal epithelial cells, perturbation of this pathway altered free fatty acid availability, 4-hydroxynonenal accumulation, TFRC expression, and ferroptotic injury [11]. These findings identify selective lipid-droplet turnover as a pro-ferroptotic input in this corneal epithelial DED context.

Lipid-droplet depletion alone cannot show that released fatty acids enter ferroptosis-sensitive phospholipids. Once released, fatty acids can undergo mitochondrial oxidation, re-esterification, storage, or signaling rather than enter membrane phospholipids directly. Isotope tracing and lipidomic analyses are needed to demonstrate their transfer into ACSL4-dependent oxidized phosphatidylethanolamine species. Such experiments should also determine whether lipophagy inhibition preserves epithelial viability without disrupting physiological lipid handling, particularly in lipid-rich meibomian tissue.

The effect of lipophagy depends on lipid composition and cellular fatty acid oxidation capacity. Lipid mobilization is more likely to be adaptive when released substrates are oxidized or re-esterified, but becomes potentially harmful when mitochondrial function is impaired and ACSL4 directs polyunsaturated fatty acids into oxidation-sensitive membrane phospholipids. Lipid-droplet turnover, acyl-chain composition, mitochondrial oxidation, and oxidized membrane phospholipids should therefore be measured within the same experimental system.

### 3.3. Impaired Autophagic Flux Promotes SQSTM1/p62–ACSL4-Associated Lipid Peroxidation

Impaired autophagic flux is mechanistically distinct from selective cargo degradation. When autophagic degradation is incomplete, damaged proteins and organelles persist, and SQSTM1/p62 accumulates. In corneal epithelial cells exposed to desiccating stress, SQSTM1/p62 accumulation associated with autophagy impairment promoted ACSL4 expression, lipid peroxidation, and ferroptotic injury [15]. These findings directly link impaired autophagic processing to increased ferroptotic susceptibility through the SQSTM1/p62–ACSL4 axis in this model.

SQSTM1/p62 does not exert a uniformly pro- or anti-ferroptotic effect. In non-ocular systems, increased SQSTM1/p62 can sequester Kelch-like ECH-associated protein 1 (KEAP1), stabilize nuclear factor erythroid 2-related factor 2 (NRF2), and activate antioxidant and anti-ferroptotic programs [58]. By contrast, the DED study links SQSTM1/p62 accumulation to increased ACSL4 expression, lipid peroxidation, and ferroptotic injury [15]. These divergent effects likely reflect differences in flux status, interacting proteins, stress intensity, and cell identity. Resolving them requires assessment of lysosomal degradative function, SQSTM1/p62 distribution and turnover, NRF2 activity, ACSL4 abundance, and the phospholipid species undergoing oxidation.

Increased SQSTM1/p62 can result from impaired degradation, transcriptional induction, or both. In the DED model, pathway perturbation linked SQSTM1/p62 accumulation to ACSL4-dependent lipid peroxidation and ferroptotic injury [15]. Epistasis experiments restoring SQSTM1/p62 or ACSL4 after flux recovery would further clarify the order of events.

## 4. Protective Effects of Restored Autophagic Homeostasis on Ferroptotic Susceptibility in DED

The opposing side of the balance comprises processes that remove damaged cargo, preserve mitochondrial quality, and support membrane peroxide detoxification. Studies in DED models associate these processes with improved ocular-surface homeostasis, although their relationship with ferroptosis differs among the pathways summarized in Table 1.

### 4.1. PINK1/Parkin-Dependent Mitophagy, Mitochondrial ROS, and Ferroptotic Priming

Damaged mitochondria can increase ROS production, disrupt cellular redox and GSH homeostasis, and release mitochondrial components that amplify inflammation. The PINK1–Parkin pathway, in which PINK1 activates the E3 ubiquitin ligase Parkin, selectively targets dysfunctional mitochondria for mitophagic clearance and thereby limits upstream oxidative pressure [59]. Melatonin-loaded copolymer micelles and cyclic arginine–glycine–aspartic acid (cRGD)-conjugated bilirubin nanoparticles improve experimental ocular-surface injury in association with PINK1-dependent mitophagy [7,47]. PINK1/Parkin-mediated mitochondrial quality control also protects aging lacrimal-gland acinar cells from mitochondrial dysfunction [53].

These studies identify mitophagy as a protective mechanism in DED-related models, but they did not determine whether ferroptosis suppression was required for protection. Combining PINK1 or Parkin perturbation with oxidized-phospholipid measurements and ferroptosis-selective rescue would resolve this relationship.

Mitochondria are not required for ferroptosis execution in every cellular context [5,6]. Nevertheless, mitochondria modify ferroptotic susceptibility through ROS generation, iron–sulfur cluster metabolism, fatty acid oxidation, and reducing-equivalent supply. Their contribution in energy-demanding lacrimal acinar cells and other ocular compartments requires tissue-specific testing. Tissue-resolved studies should determine whether mitophagy directly reduces membrane phospholipid peroxidation or primarily preserves secretion and limits the release of pro-inflammatory mitochondrial signals.

### 4.2. Intact Autophagic Flux Supports Cellular Clearance and May Restrain Ferroptotic Injury

Intact autophagic flux supports the clearance of damaged organelles and protein aggregates and limits SQSTM1/p62 accumulation [12,39]. Across DED models, pharmacological or genetic interventions that restore or enhance autophagy-related processes are associated with improved epithelial integrity and tear secretion, together with reduced oxidative stress and inflammation [43,44,45,46,47,48,49,50,51,52]. The corneal SQSTM1/p62–ACSL4 study demonstrates how autophagy impairment can promote ferroptotic injury [15]. Together, these findings provide a mechanistic rationale for restoring autophagic pathway completion in DED contexts where impaired flux or lysosomal dysfunction has been demonstrated.

This rationale argues against indiscriminate activation of autophagy. Increasing pathway initiation while downstream lysosomal degradation remains impaired may enlarge the pool of undegraded autophagosomes and cargo. Conversely, suppressing autophagy to limit ferritinophagy or lipophagy may compromise mitochondrial and protein quality control. Therapeutic interpretation should therefore be based on tandem-reporter flux, lysosomal competence, and selective cargo turnover rather than static autophagy markers alone.

Whether flux restoration suppresses ferroptosis directly should be tested by combining lysosomal-flux recovery with oxidized-phospholipid measurements, ferroptosis-selective rescue, and renewed flux blockade.

### 4.3. Autophagy-Associated Stress Signaling Reinforces Antioxidant Defense

Ferroptosis defense is mediated principally by the SLC7A11–GSH–GPX4 axis, which detoxifies phospholipid hydroperoxides, and the parallel FSP1–coenzyme Q system, which suppresses lipid-radical propagation [5,27,28,29]. DED studies support the involvement of SLC7A11/GPX4 signaling, the sirtuin 1 (SIRT1)–hypoxia-inducible factor 1α (HIF-1α)–GPX4 axis, and NRF2-related antioxidant responses [7,10,34]. Broader ferroptosis research identifies NRF2 as an important transcriptional regulator of antioxidant, iron-handling, and ferroptosis-defense genes [5,60]. Astaxanthin improves DED-associated outcomes together with autophagy-related changes and increased SLC7A11/GPX4 signaling [34]. Because these effects were measured in parallel, they do not place autophagy upstream of GPX4.

Autophagy-associated signaling also influences antioxidant capacity. In non-ocular systems, SQSTM1/p62–KEAP1–NRF2 signaling reinforces antioxidant and anti-ferroptotic defense [58], while clearance of damaged mitochondria reduces upstream oxidative pressure and supports GSH-dependent peroxide detoxification [59]. Whether these mechanisms operate at the ocular surface in DED remains unresolved.

FSP1 is an established ferroptosis suppressor acting in parallel to GPX4 [5,28,29], but its role in DED is largely unexplored. GPX4 and FSP1 are membrane-defense systems rather than components of the autophagic machinery. Their functional coordination with autophagy should be tested by perturbing autophagic flux while measuring the activity and rescue capacity of each antioxidant pathway.

### 4.4. Contextual Determinants of the Ferroptosis Threshold Balance

The available evidence can be organized as a threshold balance between pro-ferroptotic pressure and clearance-defense capacity. In the indicated epithelial models, NCOA4-dependent ferritinophagy and ATG5-dependent lipophagy can increase the availability of iron and lipid substrates. Impaired downstream lysosomal degradation allows damaged cargo to accumulate and promotes SQSTM1/p62–ACSL4 signaling. In contrast, intact autophagic flux and mitophagy reduce damaged cargo and oxidative burden, whereas GPX4- and FSP1-related systems protect membranes from phospholipid peroxidation. Ferroptotic injury becomes more likely when substrate loading and lipid-peroxidation pressure exceed these combined defenses.

Four variables shift this balance: flux integrity, selective cargo, stress burden, and tissue compartment. The effect of an autophagic pathway therefore differs across biological contexts. NCOA4 inhibition is most relevant when ferritin turnover expands the labile iron pool; lipophagy inhibition is most relevant when lipid-droplet-derived fatty acids enter oxidation-sensitive membrane phospholipids; and restoration of lysosomal function is most relevant when impaired flux completion predominates.

**Figure 3:**
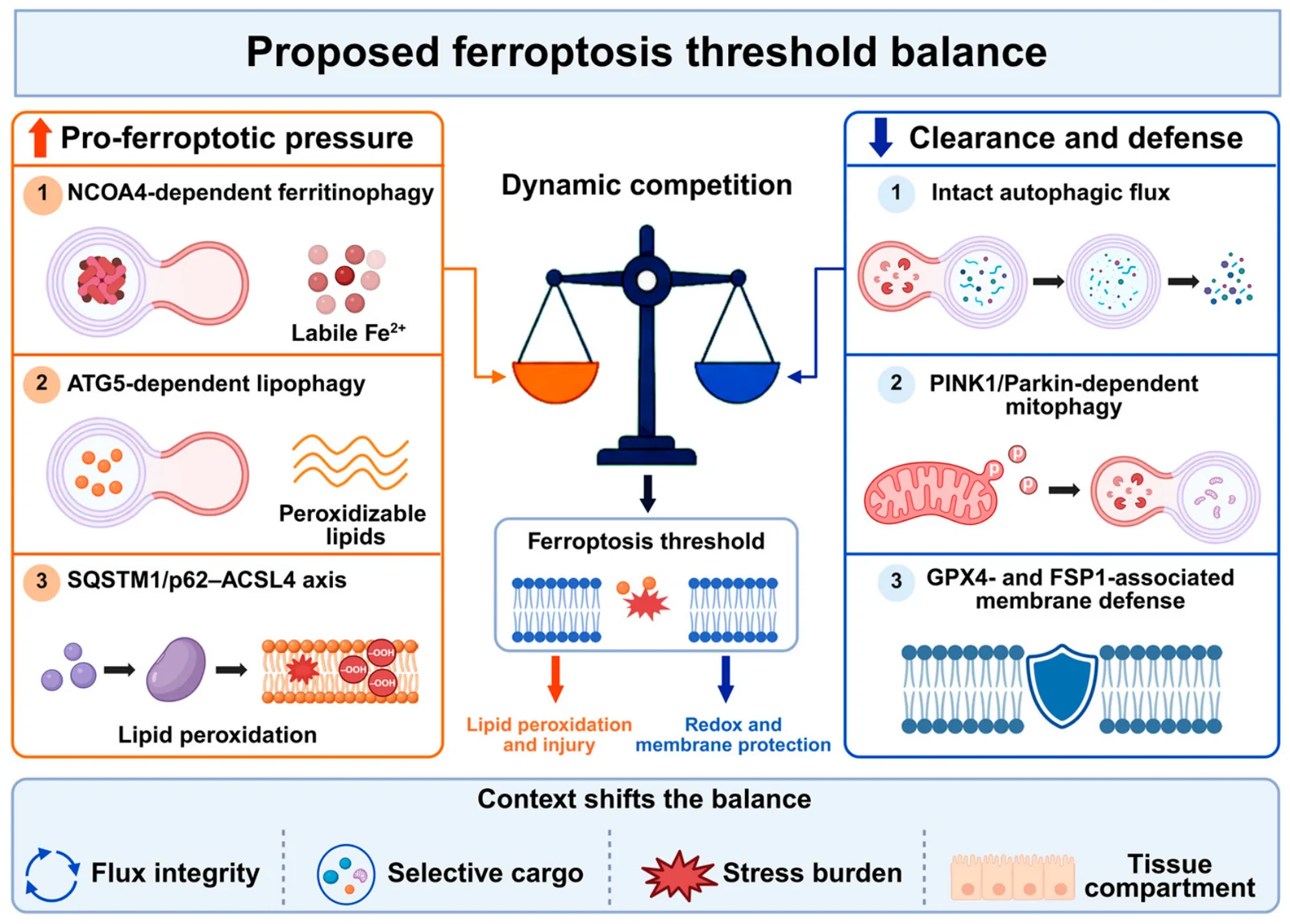
Proposed threshold-balance model of autophagy-related regulation of ferroptotic susceptibility in dry eye disease. The model integrates opposing autophagy-related and membrane-defense processes. NCOA4-dependent ferritinophagy expands the labile iron pool, ATG5-dependent lipophagy mobilizes peroxidizable lipid substrates, and impaired autophagic flux promotes SQSTM1/p62–ACSL4-associated phospholipid peroxidation in the indicated experimental models. On the opposing side, intact autophagic flux and PINK1/Parkin-dependent mitophagy are positioned to remove damaged cargo and dysfunctional mitochondria, whereas GPX4- and FSP1-associated systems limit membrane phospholipid peroxidation. The –OOH symbols represent phospholipid hydroperoxide groups generated during lipid peroxidation. The proposed balance varies with flux integrity, selective cargo, stress burden, and tissue compartment and provides a framework for determining whether stressed cells maintain homeostasis or progress toward ferroptotic injury. ACSL4, acyl-CoA synthetase long-chain family member 4; ATG5, autophagy-related protein 5; Fe^2+^, ferrous iron; FSP1, ferroptosis suppressor protein 1; GPX4, glutathione peroxidase 4; NCOA4, nuclear receptor coactivator 4; P, phosphorylation; PINK1, PTEN-induced kinase 1; SQSTM1/p62, sequestosome 1.

Figure 3 presents this framework as a qualitative and testable model. It does not assign fixed weights to individual pathways, define a numerical threshold, or imply that all components have been demonstrated within a single DED model. Rather, it identifies the measurements required to determine which side of the balance predominates in a defined cell type and stress context.

Tissue compartment modifies both sides of the balance. Corneal and conjunctival epithelia are directly exposed to environmental stressors and require rapid barrier repair [1,2]. Lacrimal acinar cells require intact mitochondrial function to support energy-intensive protein and fluid secretion [53,54]. Meibomian-gland progenitor cells reside within a lipid-metabolically specialized gland, and ferroptotic injury to these cells can contribute to experimental meibomian gland dysfunction [37]. The same intervention may therefore protect one compartment while disrupting homeostasis in another. Whole-eye outcomes should be paired with cell-specific measurements of target engagement, pathway completion, and ferroptotic injury.

## 5. Dynamic Autophagy–Ferroptosis Crosstalk in Ocular-Surface Injury and Homeostatic Failure

**Figure 4:**
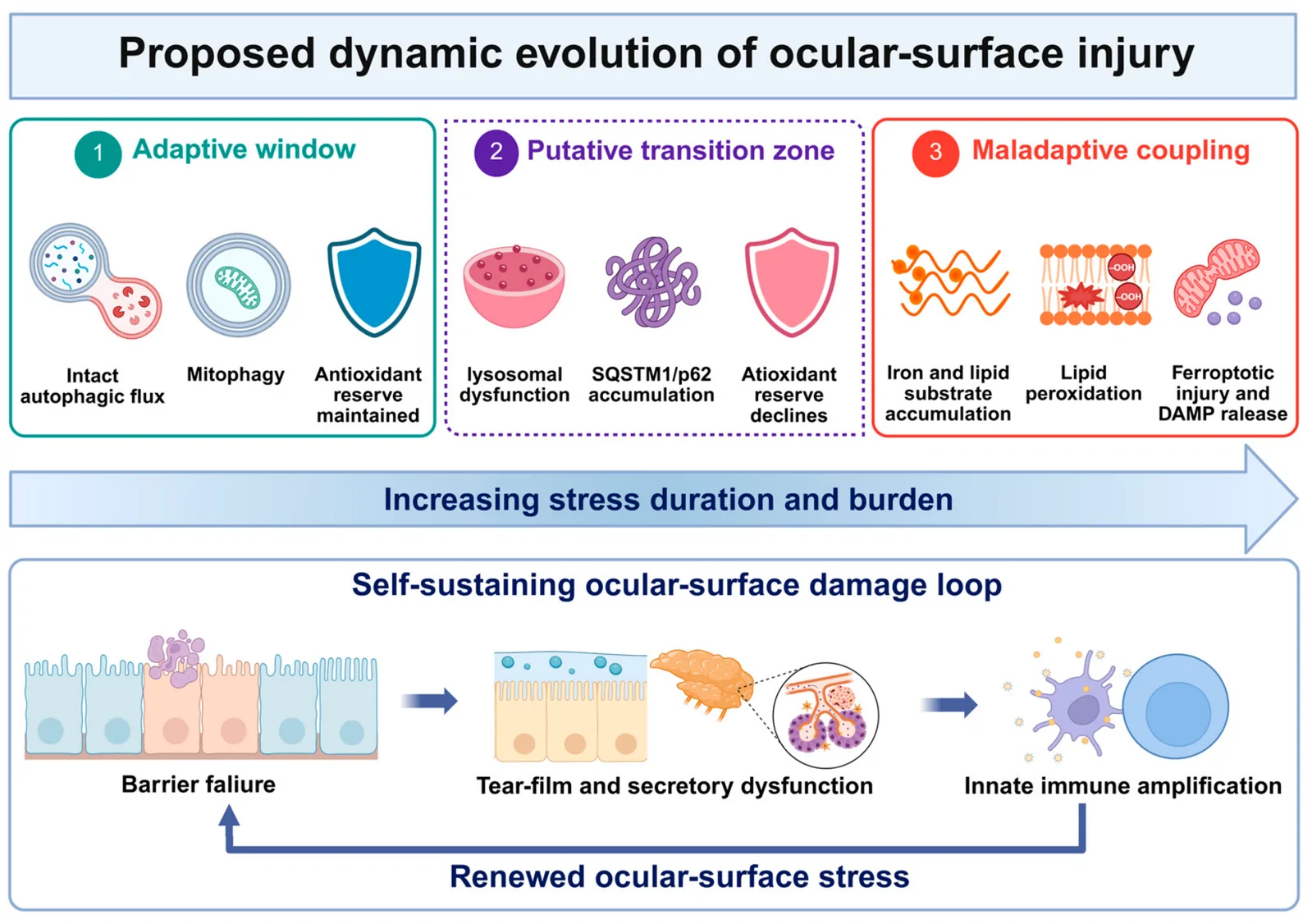
Proposed dynamic evolution of autophagy–ferroptosis crosstalk during ocular-surface injury. During the proposed adaptive window, intact autophagic flux, mitophagy, and antioxidant capacity maintain a state of relatively low ferroptotic susceptibility. With increasing stress duration and burden, lysosomal dysfunction, SQSTM1/p62 accumulation, and declining antioxidant reserves define a putative transition zone. Further accumulation of iron and peroxidizable lipid substrates is proposed to promote phospholipid peroxidation, ferroptosis-associated injury, and DAMP release. The –OOH symbols denote phospholipid hydroperoxide groups generated during lipid peroxidation. At the tissue level, epithelial barrier failure, tear-film and secretory dysfunction, and innate immune amplification form a proposed feedback loop that renews hyperosmolar, oxidative, and inflammatory stress. Green, purple, and red frames denote the adaptive window, putative transition zone, and maladaptive coupling, respectively. Rightward arrows indicate the proposed direction of progression with increasing stress, whereas the return arrow denotes renewed stress within the feedback loop. The three stages constitute a testable framework rather than an established temporal sequence, biological staging system, or clinical classification. DAMPs, damage-associated molecular patterns; SQSTM1/p62, sequestosome 1.

At the level of the lacrimal functional unit, the threshold balance is associated with three interconnected consequences: epithelial barrier injury, tear-film and secretory dysfunction, and innate immune amplification. Their sequence depends on the initiating stressor and affected ocular compartment. Figure 4 integrates these relationships into a testable model rather than a universal disease timeline.

### 5.1. Ferroptosis-Associated Epithelial Injury and Barrier Integrity

Corneal and conjunctival epithelia are directly exposed to hyperosmolarity, evaporation, pollutants, and light-associated stress [8,20,21,22,23,24]. In these cells, NCOA4-dependent ferritinophagy, ATG5-dependent lipophagy, and autophagy impairment associated with SQSTM1/p62 accumulation can increase iron availability or lipid-peroxidation pressure [8,11,15]. When GPX4-related defense becomes insufficient, phospholipid peroxidation can compromise membrane integrity and epithelial viability [6,10,27,33,34].

Barrier dysfunction extends the consequences beyond the loss of individual cells. Disruption of intercellular junctions and impaired epithelial repair alters surface wettability, exposes sensory and immune interfaces, and increases susceptibility to environmental stressors. Direct links between autophagy–ferroptosis coupling and these tissue-level barrier outcomes remain to be tested.

Future studies should link autophagy-dependent changes in ferroptotic susceptibility to transepithelial permeability, junctional protein organization, wound closure, and corneal staining. These functional endpoints would help distinguish a tissue-level pathogenic mechanism from an association based only on intracellular markers.

### 5.2. Lacrimal- and Meibomian-Gland Dysfunction Destabilizes Tear-Film Homeostasis

Following corneal nerve severing, lacrimal glands exhibit iron accumulation, ferroptosis-associated molecular changes, acinar injury, and reduced tear secretion [9]. Autophagy and mitophagy also support mitochondrial quality control and secretory homeostasis in lacrimal acinar-cell and organoid models [53,54]. These findings place ferroptosis-associated lacrimal injury and autophagic homeostasis within the same broad tissue compartment, but the two processes have not yet been connected within a single causal experiment.

Related salivary-gland studies in Sjögren syndrome provide additional mechanistic context. GPX4 loss and lipid ROS accumulation contribute to salivary epithelial dysfunction and impaired secretion [61], whereas restoration of autophagy improves salivary-gland function and inflammatory pathology in non-obese diabetic mice [62]. More recent work further indicates that targeting TFRC/FTH1/NCOA4-associated ferritinophagy can restore antioxidant defense and alleviate salivary-gland dysfunction [63]. These salivary-gland studies inform the lacrimal hypothesis but do not establish the same mechanism in DED.

Meibomian glands present a distinct challenge because they are highly specialized for lipid synthesis and secretion. Ferroptosis in meibomian-gland progenitor cells contributes to experimental meibomian gland dysfunction [37], whereas ATG5-dependent lipophagy mobilizes peroxidizable lipid substrates in corneal epithelial DED [11]. Autophagy–ferroptosis coupling in the meibomian gland remains untested, although its demonstrated ferroptotic susceptibility is relevant to evaporative DED.

### 5.3. Mitochondrial Damage-Associated Molecular Patterns Amplify Innate Immune Signaling

Mitochondrial damage-associated molecular patterns (DAMPs) may connect ferroptosis-associated stress with sterile inflammation. In DED-relevant corneal epithelial and ocular-surface models, oxidized mitochondrial DNA (mtDNA) or cytosolic mtDNA leakage activates NLR family pyrin domain containing 3 (NLRP3) or absent in melanoma 2 (AIM2) inflammasome signaling [64,65]. In lacrimal-gland myoepithelial cells, cytosolic genomic DNA activates the AIM2 inflammasome and stimulator of interferon genes (STING) signaling [66].

Mechanistic studies outside DED further show that selective autophagy can clear leaked cytosolic mtDNA [67], whereas core autophagy proteins can limit mitochondrial damage, mtDNA release, and downstream inflammasome activation [68]. A complementary macrophage study showed that maintenance of SQSTM1/p62 expression restricts mitochondrial damage, mtDNA release, and NLRP3 inflammasome activation [69]. Selective autophagy can also mediate SQSTM1/p62-dependent turnover of STING and thereby restrain cyclic GMP–AMP synthase (cGAS)–STING signaling [70]. In DED models, cyclosporine A suppresses cGAS–STING signaling in association with autophagy induction [43], whereas chrysin reduces ferroptosis-associated injury and STING/NLRP3-associated inflammatory responses [32].

Together, these findings identify convergence among mitochondrial injury, autophagic quality control, ferroptosis-associated stress, and innate immune signaling. The order and interdependence of these events remain unresolved.

Autophagic degradation of retinoic acid-inducible gene I (RIG-I) also limits type I interferon signaling in human conjunctival epithelial cells [71], illustrating the broader immunoregulatory role of autophagy. Combining ferroptosis-selective rescue with measurements of mtDNA release, inflammasome or STING activation, and ocular-surface inflammation would determine whether ferroptotic injury drives inflammatory amplification or occurs in parallel with it.

### 5.4. Maladaptive Coupling and the Self-Sustaining Damage Loop

During early stress exposure, intact autophagic flux, regulated mitophagy, and antioxidant defense support a state of low ferroptotic susceptibility [72,73,74]. Persistent or severe stress disrupts lysosomal homeostasis, reduces antioxidant reserve, and increases iron and lipid-substrate pressure [10,75]. Lipid-peroxidative injury to epithelial and secretory tissues is then positioned to aggravate tear-film instability and inflammation [9,30,31,32,33,34,76,77], renewing hyperosmolar and oxidative stress [3,4,21]. This proposed feedback links adaptive stress responses to maladaptive autophagy–ferroptosis coupling.

Figure 4 presents an adaptive window, a putative transition zone, and maladaptive coupling as a hypothesis derived from the integrated evidence. No study has established a universal transition time or shown that every DED model follows this sequence. The model is intended for experimental testing rather than as an established natural history of DED.

Time-resolved studies should distinguish early adaptation from later pathway failure and determine whether autophagic-flux impairment, selective cargo turnover, mitochondrial dysfunction, antioxidant depletion, and phospholipid peroxidation arise sequentially or in parallel.

Reversibility is another important feature of the proposed model. A transition zone is therapeutically meaningful only if restoring autophagic flux or antioxidant capacity can return cells to a state of lower ferroptotic susceptibility [72,74,76,77]. Delayed intervention at multiple time points, followed by treatment withdrawal and recurrence assessment, could distinguish transient biochemical improvement from durable interruption of the damage loop. Such experiments would be more informative than comparisons restricted to untreated and prophylactically treated endpoints.

## 6. Mechanism-Guided Translational Implications and Validation Priorities

Translation requires matching each intervention to a defined mechanistic defect. Current therapeutic evidence is predominantly preclinical, and the clinical benefit of tacrolimus has not been linked to autophagy–ferroptosis coupling. Molecular stratification and nonspecific modulation of autophagy are therefore not yet justified. Immediate priorities include target engagement, ocular safety, and functional validation in well-defined DED models.

### 6.1. Restoring Functional Autophagic Flux to Preserve Ocular-Surface Homeostasis

Restoration of autophagic flux is most defensible when impaired autophagosome–lysosome fusion or lysosomal degradation has been demonstrated [15,39]. Cyclosporine A, salidroside, evodiamine, melatonin, and tacrolimus, together with activation of sirtuin 3 (SIRT3), inhibition of serum- and glucocorticoid-regulated kinase 1 (SGK1), and inhibition of euchromatic histone-lysine N-methyltransferase 2 (EHMT2/G9a), improve outcomes in experimental DED and related ocular-surface models in association with autophagy-related changes and, in some studies, improved autophagy–lysosomal function [43,44,45,46,47,49,50,51]. Tacrolimus has shown clinical benefit [50], although its proposed autophagy–lysosomal mechanism has not been linked to ferroptosis suppression.

Validation should begin with direct evidence of lysosomal dysfunction, followed by tandem fluorescent reporters, lysosomal-inhibitor experiments, or cargo-turnover assays to confirm pathway completion [39]. Flux restoration should then be linked to reduced SQSTM1/p62–ACSL4 signaling, oxidized phospholipids, and ferroptosis-related injury, with ferroptosis-selective rescue used to test pathway specificity [15]. This sequence separates flux-dependent effects from independent antioxidant or anti-inflammatory activity.

Intervention timing is equally important. Increasing autophagy initiation may be ineffective or harmful when lysosomal degradation remains impaired [15,39]. Flux-restoring treatments need evaluation at different stages of stress exposure and comparison with interventions that increase initiation without correcting lysosomal function.

### 6.2. Rebalancing Selective Cargo Handling to Reduce Ferroptotic Susceptibility

When ferritinophagy expands the labile iron pool, local NCOA4 inhibition may reduce pro-ferroptotic pressure [8,55,56,57]. Iron chelation represents a related therapeutic possibility, but its efficacy and ocular safety require direct validation in DED models. When lipophagy supplies oxidizable lipid substrates, selective control of lipid-droplet mobilization may be more appropriate [11]. Both strategies require precise targeting because iron and lipid turnover are essential for epithelial metabolism, mitochondrial function, and meibomian differentiation.

Candidate interventions should be selected according to the dominant cargo pathway rather than a generic ferroptosis-associated signature. Ferritin degradation accompanied by lysosomal dependence and increased labile Fe^2+^ would support an iron-supply mechanism [8,55,56,57]. Lipid-droplet depletion accompanied by transfer of fatty acids into ACSL4-dependent oxidized phospholipids would support a lipid-supply mechanism [11,15,26]. These profiles are experimental selection criteria, not validated clinical endotypes.

Therapeutic studies should also distinguish pathway-selective effects from general suppression of autophagy. Inhibition of NCOA4-dependent ferritinophagy should be tested against broader changes in iron uptake, export, and storage. Similarly, inhibition of lipophagy should be evaluated for effects on physiological lipid turnover and mitochondrial metabolism. This is particularly important in meibomian tissues, where lipid handling is integral to normal differentiation and secretion [37].

### 6.3. Reinforcing GPX4- and FSP1-Related Redox Defense to Limit Lipid Peroxidation

Strategies that restore cystine uptake, GSH availability, or GPX4 activity act close to the execution phase of ferroptosis [6,27,34,74]. The FSP1–coenzyme Q defense system provides a complementary GSH-independent pathway [28,29], although direct ocular-surface evidence remains limited. Reinforcing membrane antioxidant defense offers an alternative when iron and lipid-substrate pressure cannot be reduced safely or selectively.

Many candidate compounds are pleiotropic. Mechanistic studies need to separate direct radical-trapping effects from changes in SLC7A11, GPX4, FSP1, autophagic flux, and mitochondrial quality control. Rescue and loss-of-function experiments are needed to establish which pathway is necessary for protection. Changes in antioxidant-protein abundance alone are insufficient to demonstrate target engagement.

Combination therapy is justified only after each component demonstrates independent target engagement and acceptable ocular safety. The possibility of pharmacological redundancy, antagonism, or disruption of physiological redox signaling should also be evaluated. These requirements are especially important for prolonged topical treatment of a chronically exposed epithelium.

### 6.4. Limiting Ferroptosis-Associated Inflammatory Amplification

Interventions that preserve mitochondrial integrity or limit mtDNA oxidation may reduce inflammasome activation in DED models [20,64,65]. Autophagic regulation of inflammasomes and cGAS–STING signaling provides a mechanistic rationale for targeting this axis [67,68,69,70]. Other approaches, including suppression of ferroptosis-associated STING/NLRP3 activation or broader ocular-surface inflammatory signaling, remain supported mainly by preclinical evidence [32,64,74]. Anti-inflammatory efficacy alone is not evidence of ferroptosis specificity. Ferroptosis-selective rescue should therefore be tested against inflammatory outcomes while ferroptotic injury is experimentally controlled.

This distinction is important because inflammation in DED has multiple initiating mechanisms [2,3,4]. A mechanism-guided approach must establish whether ferroptotic epithelial or glandular injury contributes measurably to cytokine release, immune-cell recruitment, and recurrent tear-film instability; it cannot assume that all inflammatory activity lies downstream of ferroptosis.

Temporal analysis is particularly important for this pathway. Temporal profiling can establish whether ferroptotic injury precedes, accompanies, or follows innate immune activation. Without that sequence, interruption of either process cannot be assumed to disrupt the entire feedback loop.

### 6.5. Tissue-Specific Delivery, Biomarkers, and Human Validation

Topical administration is attractive for targeting the corneal and conjunctival epithelia, but short ocular residence time, limited epithelial penetration, formulation instability, and reflex tearing remain substantial barriers. Lacrimal-gland and deeper meibomian-gland pathology may therefore require alternative delivery strategies. Mucin-targeted liposomes, micelles, and nanoparticle-based systems have improved ocular retention, local exposure, or intracellular delivery in experimental DED models [7,33,47,64,77,78]. Each platform still requires evaluation of tissue distribution, cellular target engagement, ocular tolerability, and functional benefit.

Human validation needs to connect accessible molecular measurements with clinically relevant ocular function. Tear samples can support assessment of tear-protein alterations, lipidomic features, antioxidant-related components, and inflammatory mediators [79,80,81], whereas impression cytology can provide ocular-surface cells for selected gene-expression and molecular analyses [82]. Neither approach alone, however, can establish intracellular autophagic flux or ferroptotic cell death [5,6,39]. Candidate biomarkers therefore need to be anchored to flux-aware assays in ex vivo tissues, organoids, or other experimentally validated, tissue-relevant models.

Biomarker assessment should distinguish stress exposure, mechanistic activity, and tissue-level consequences. Tear osmolarity and environmental history can characterize stress exposure [1]. Tear protein and lipid profiles may provide candidate indicators of oxidative, metabolic, or inflammatory activity [79,80,81]. Cellular iron handling, ferritin turnover, SQSTM1/p62 accumulation, lysosomal competence, and oxidized phospholipids remain exploratory mechanistic readouts rather than validated clinical biomarkers.

Candidate molecular markers should be evaluated alongside endpoints appropriate to the affected tissue, including tear-film stability, tear secretion, epithelial permeability, corneal staining, corneal sensitivity, goblet-cell density, and meibomian-gland structure [1,37,79,80,81,82]. Longitudinal studies should establish analytical validity, tissue relevance, temporal associations, and relationships with ocular function before biomarker panels are used for patient stratification, treatment selection, or response prediction.

## 7. Evidence Gaps and Future Directions

### 7.1. Establishing Causal Autophagy–Ferroptosis Crosstalk

Future studies should move beyond parallel endpoint measurements by perturbing defined autophagic components and determining the resulting changes in ferroptotic susceptibility. Reciprocal experiments should test whether ferroptosis induction or inhibition alters autophagic flux or selective cargo turnover.

Using the mechanistic criteria defined in Section 2.1 and Section 2.2, genetic or pharmacological epistasis experiments should determine whether a specific autophagic pathway acts upstream, downstream, or independently of ferroptosis.

Time-resolved experiments are also needed because current evidence does not support a universal temporal sequence across DED models. Sampling must establish the order of flux impairment, ferritin or lipid-droplet turnover, mitochondrial dysfunction, antioxidant depletion, and phospholipid peroxidation. Because this sequence may differ among hyperosmolar, desiccating-stress, cigarette-smoke, blue-light, corneal-denervation, meibomian-gland dysfunction, and autoimmune models, transition points must be defined separately for each stressor, tissue compartment, and phase of experimental injury.

### 7.2. Resolving Cell- and Tissue-Specific Mechanisms

Much of the available evidence is derived from corneal epithelial cultures and relatively short-duration animal models [13,14,15,16,17,18,19]. Other components of the lacrimal functional unit, including lacrimal and meibomian tissues, remain less completely characterized [18,20,37,53,54]. Conjunctival goblet cells, lacrimal acinar cells, meibomian-gland cells, sensory neurons, and resident immune cells differ substantially in their metabolic demands. They may also differ in iron handling, lipid composition, mitochondrial dependence, and lysosomal capacity. Resolving these differences will require cell-specific genetic models, organoids, spatial profiling, and ex vivo human tissues.

Model selection should be aligned with the mechanism and DED phenotype under investigation. Hyperosmolar and desiccating-stress models primarily reproduce consequences of tear-film instability, whereas cigarette smoke and blue light represent environmental injury. Corneal-denervation models are suited to studying neural–lacrimal dysfunction, while meibomian-gland models address lipid-rich secretory tissues. Autoimmune models are more appropriate for chronic inflammatory and glandular injury. A mechanism identified in one model should not be generalized across DED phenotypes without replication in the relevant tissues and disease contexts.

### 7.3. Human Validation and Functional Endpoints

Human validation must address both biological heterogeneity and methodological reproducibility. Stratification by aqueous-deficient, evaporative, autoimmune, environmental, and neurotrophic phenotypes may distinguish shared mechanisms from phenotype-specific changes [1,2,3,4,38]. Cross-cohort comparison of candidate biomarkers depends on standardized sample collection, processing, storage, and analysis.

Molecular target engagement must ultimately be linked to clinically meaningful ocular outcomes and validated symptom measures [1,37,79,80,81,82]. Tissue-based assays, ex vivo functional studies, and delayed-treatment, durability, and recurrence designs are needed to determine whether autophagy–ferroptosis modulation produces sustained functional benefit. These advances are prerequisites for biomarker-guided patient stratification or therapeutic selection in DED.

## 8. Conclusions

The available evidence supports pathway-specific effects of autophagy on ferroptotic susceptibility in DED. NCOA4-dependent ferritinophagy and ATG5-dependent lipophagy can increase access to redox-active iron and peroxidizable lipid substrates, whereas impaired autophagic flux promotes SQSTM1/p62 accumulation and ACSL4-associated phospholipid peroxidation in established epithelial models. In contrast, intact autophagic–lysosomal flux and mitophagy limit the accumulation of damaged cargo and dysfunctional mitochondria. Their proposed coordination with GPX4- and FSP1-associated defenses could restrain ferroptotic injury.

Studies combining autophagy-related pathway perturbation with ferroptosis-related outcomes are currently concentrated in corneal epithelial models and in a blue-light-exposed conjunctival epithelial model. In lacrimal and meibomian tissues and in models of inflammatory amplification, autophagy and ferroptosis have more often been examined separately, and their mechanistic relationship remains insufficiently tested. The threshold-balance and dynamic damage-loop models should therefore be regarded as testable mechanistic syntheses rather than established biological sequences or clinical classifications. Progress will require flux-aware pathway perturbation, ferroptosis-selective rescue, tissue-resolved models, and validation in human tissues and clinically relevant functional outcomes. These approaches are needed to establish whether autophagy–ferroptosis crosstalk can support biomarker development or mechanism-guided treatment of DED.

## Figures and Tables

**Table 1:** Qualitative map of autophagy, ferroptosis, and their investigated relationships in DED-relevant models.

Mechanistic Module	Ferroptosis-Related Criteria Assessed	Principal Model or Tissue	Main Finding or Mechanistic Inference	Relationship Evaluated	Key Limitation	Reference
NCOA4-dependent ferritinophagy	Iron accumulation, lipid peroxidation, ferroptosis-related injury, and NCOA4 perturbation	Blue-light-exposed conjunctival epithelium	Ferritin degradation expands the labile iron pool and increases ferroptotic susceptibility	Direct mechanistic evidence: autophagy-related pathway perturbation altered ferroptosis-related injury	Evidence derives from an exposure-specific model and requires independent replication	[8]
SQSTM1/p62–ACSL4 axis	ACSL4-dependent lipid peroxidation, ferroptosis-related injury, and SQSTM1/p62 perturbation	Desiccating-stressed corneal epithelium	Incomplete autophagic degradation promotes ACSL4-associated phospholipid peroxidation and ferroptotic injury	Direct mechanistic evidence: SQSTM1/p62 perturbation was assessed together with ACSL4-dependent lipid peroxidation and ferroptosis-related injury	Evidence remains concentrated in the corneal epithelium	[15]
ATG5-dependent lipophagy	Lipid peroxidation markers, TFRC expression, ferroptosis-related injury, and ATG5-dependent pathway perturbation	Corneal epithelial DED models	Lipid-droplet mobilization increases the availability of ferroptosis-sensitive lipid substrates	Direct mechanistic evidence: ATG5-dependent lipophagy was perturbed together with assessment of ferroptosis-related injury	Transfer of mobilized fatty acids into oxidized membrane phospholipids requires further validation	[11]
Ferroptosis inhibition and autophagic-flux recovery	Ferroptosis-related injury, SLC7A11–GSH–GPX4 defense, and ferroptosis-selective rescue; tandem-reporter autophagic-flux assessment was also performed	Hyperosmolarity-exposed corneal epithelial cells and experimental DED	Ferroptosis inhibition was accompanied by autophagy-related changes, and astaxanthin restored tandem-reporter autophagic flux	Associative/one-directional evidence: ferroptosis inhibition was followed by flux recovery; autophagy dependence was not tested	Autophagy-specific perturbation was not used to determine whether flux recovery was required for ferroptosis suppression or ocular protection	[34]
Ferroptosis in meibomian-gland progenitor cells	Ferroptosis-related injury in meibomian-gland progenitor cells; autophagy was not assessed	Experimental meibomian-gland dysfunction	Ferroptosis in progenitor cells contributes to experimental meibomian-gland dysfunction.	Single-process evidence: ferroptosis was assessed, but autophagy involvement was not tested	A causal autophagic cargo pathway has not been established	[37]
Mitophagy and lacrimal-gland quality control	Ferroptosis was not directly assessed in the autophagy/mitophagy studies summarized in this row	Hyperosmolar ocular-surface models, lacrimal acinar cells, and gland organoids	Mitochondrial quality control limits oxidative injury and supports secretory homeostasis	Single-process or contextual evidence: autophagy or mitophagy was assessed, but coupling to ferroptosis was not tested	Mitophagy perturbation and ferroptosis-selective rescue have not been consistently combined	[7,47,53,54]
Broad autophagy–lysosomal regulation	Ferroptosis was not directly assessed in the DDIT4 or MIATNB flux studies	Corneal epithelial cells, conjunctival epithelial cells, and experimental DED models	Autophagy-related regulation is associated with ocular-surface protection; DDIT4 impairs, whereas MIATNB supports, autophagic flux under hyperosmolar stress	Single-process evidence: autophagic function or flux was assessed, but ferroptosis coupling was not tested	Flux validation varies among studies, and neither the DDIT4 nor the MIATNB study assessed iron-dependent phospholipid peroxidation or ferroptosis-selective rescue	[41,42,43,44,45,46,47,49,50,51,52]

Note: This table provides a qualitative map and is not intended as a formal evidence-grading system. Direct mechanistic evidence denotes perturbation of a defined autophagy-related component together with a change in ferroptosis-related injury. Associative or one-directional evidence denotes parallel assessment of both processes or perturbation of one process followed by a change in the other without reciprocal pathway testing. Single-process evidence denotes studies that assessed only autophagy, mitophagy, or ferroptosis within DED-relevant models, whereas contextual evidence refers to non-ocular or related exocrine studies used to interpret mechanisms that remain unresolved at the ocular surface. These categories describe what was experimentally evaluated and do not establish causal crosstalk or generalizability across DED phenotypes or ocular tissues. ACSL4, acyl-CoA synthetase long-chain family member 4; ATG5, autophagy-related protein 5; DDIT4, DNA damage-inducible transcript 4; DED, dry eye disease; MIATNB, MIAT neighbor long noncoding RNA; NCOA4, nuclear receptor coactivator 4; PINK1, PTEN-induced kinase 1; SQSTM1/p62, sequestosome 1.

## Data Availability

No new data were created or analyzed in this study. Data sharing is not applicable to this article.

## Supplementary Materials

The following supporting information can be downloaded at: https://www.mdpi.com/article/10.3390/cells15171542/s1, Table S1: PubMed search strategy used for literature identification.

## Abbreviations

The following abbreviations are used in this manuscript:

ACSL4	acyl-CoA synthetase long-chain family member 4
AIM2	absent in melanoma 2
ATG5	autophagy-related protein 5
cGAS	cyclic GMP–AMP synthase
cRGD	cyclic arginine–glycine–aspartic acid
DAMPs	damage-associated molecular patterns
DDIT4	DNA damage-inducible transcript 4
DED	dry eye disease
DNA	deoxyribonucleic acid
EHMT2/G9a	euchromatic histone-lysine N-methyltransferase 2, also known as G9a
FSP1	ferroptosis suppressor protein 1
FTH1	ferritin heavy chain 1
GPX4	glutathione peroxidase 4
GSH	glutathione
HIF-1α	hypoxia-inducible factor 1α
KEAP1	Kelch-like ECH-associated protein 1
LC3	microtubule-associated protein 1 light chain 3
LC3-II	lipidated form of LC3
MIATNB	MIAT neighbor long noncoding RNA
mtDNA	mitochondrial DNA
NCOA4	nuclear receptor coactivator 4
NLRP3	NLR family pyrin domain containing 3
NRF2	nuclear factor erythroid 2-related factor 2
PINK1	PTEN-induced kinase 1
RIG-I	retinoic acid-inducible gene I
ROS	reactive oxygen species
S100A9	S100 calcium-binding protein A9
SGK1	serum- and glucocorticoid-regulated kinase 1
SIRT1	sirtuin 1
SIRT3	sirtuin 3
SLC7A11	solute carrier family 7 member 11
SQSTM1/p62	sequestosome 1, also known as p62
STING	stimulator of interferon genes
TFRC	transferrin receptor 1
TLR4	Toll-like receptor 4
YAP	Yes-associated protein
