# Supplementary material for: Autophagy–Ferroptosis Crosstalk in Dry Eye Disease: Context-Dependent Regulation of Ocular Surface Homeostasis
Source: Cells. 2026 Aug 26;15(17):1542. doi: 10.3390/cells15171542 (PMC13565532; doi:10.3390/cells15171542)
Supplement: Supplementary file 1 [file cells-15-01542-s001.zip › cells-4502601-supplementary.pdf]

Supplementary Materials

Autophagy–Ferroptosis Crosstalk in Dry Eye Disease: Context-Dependent Regulation of Ocular Surface Homeostasis

Table S1. PubMed search strategy used for literature identification

Search block	Search focus	Exact PubMed search string	Search coverage	Results
Search 1	DED and autophagy or lysosomal function	("Dry Eye Syndromes"[Mesh] OR "dry eye"[Title/Abstract] OR "dry eye disease"[Title/Abstract] OR "dry eye syndrome"[Title/Abstract] OR "keratoconjunctivitis sicca"[Title/Abstract]) AND ("Autophagy"[Mesh] OR autophag*[Title/Abstract] OR "autophagic flux"[Title/Abstract] OR lysosom*[Title/Abstract] OR autolysosom*[Title/Abstract])	Database inception to 15 July 2026; updated to 22 August 2026	181
Search 2	DED and ferroptosis or lipid peroxidation	("Dry Eye Syndromes"[Mesh] OR "dry eye"[Title/Abstract] OR "dry eye disease"[Title/Abstract] OR "dry eye syndrome"[Title/Abstract] OR "keratoconjunctivitis sicca"[Title/Abstract]) AND ("Ferroptosis"[Mesh] OR ferroptos*[Title/Abstract] OR "lipid peroxidation"[MeSH Terms] OR "lipid peroxidation"[Title/Abstract] OR "phospholipid peroxidation"[Title/Abstract] OR "iron homeostasis"[Title/Abstract] OR "iron metabolism"[Title/Abstract] OR "labile iron"[Title/Abstract] OR GPX4[Title/Abstract] OR SLC7A11[Title/Abstract] OR ACSL4[Title/Abstract] OR FSP1[Title/Abstract])	Database inception to 15 July 2026; updated to 22 August 2026	86

Search 3	Autophagy–ferroptosis relationships in DED	("Dry Eye Syndromes"[Mesh] OR "dry eye"[Title/Abstract] OR "dry eye disease"[Title/Abstract] OR "dry eye syndrome"[Title/Abstract] OR "keratoconjunctivitis sicca"[Title/Abstract]) AND (autophag*[Title/Abstract] OR "autophagic flux"[Title/Abstract] OR lysosom*[Title/Abstract] OR ferritinophag*[Title/Abstract] OR lipophag*[Title/Abstract] OR mitophag*[Title/Abstract]) AND (ferroptos*[Title/Abstract] OR "phospholipid peroxidation"[Title/Abstract] OR "lipid peroxidation"[Title/Abstract] OR GPX4[Title/Abstract] OR SLC7A11[Title/Abstract] OR ACSL4[Title/Abstract])	Database inception to 15 July 2026; updated to 22 August 2026	15
Search 4	Selective autophagy in DED-relevant ocular tissues	("Cornea"[Mesh] OR "Conjunctiva"[Mesh] OR cornea*[Title/Abstract] OR conjunctiv*[Title/Abstract] OR "corneal epithelium"[Title/Abstract] OR "conjunctival epithelium"[Title/Abstract]) AND (ferritinophag*[Title/Abstract] OR lipophag*[Title/Abstract] OR mitophag*[Title/Abstract] OR "autophagic flux"[Title/Abstract] OR NCOA4[Title/Abstract] OR ATG5[Title/Abstract] OR SQSTM1[Title/Abstract] OR p62[Title/Abstract] OR PINK1[Title/Abstract] OR Parkin[Title/Abstract] OR DDIT4[Title/Abstract] OR MIATNB[Title/Abstract]) AND ("dry eye"[Title/Abstract] OR hyperosmolar*[Title/Abstract] OR desiccat*[Title/Abstract] OR "oxidative stress"[Title/Abstract] OR "blue light"[Title/Abstract] OR "cigarette smoke"[Title/Abstract])	Database inception to 15 July 2026; updated to 22 August 2026	67
Search 5	Tissue-specific evidence from ocular-surface and glandular models	("Lacrimal Apparatus"[Mesh] OR "Meibomian Glands"[Mesh] OR "lacrimal gland"[Title/Abstract] OR "lacrimal glands"[Title/Abstract] OR "lacrimal acinar"[Title/Abstract] OR "lacrimal organoid"[Title/Abstract] OR "meibomian gland"[Title/Abstract] OR "meibomian glands"[Title/Abstract] OR meibocyte*[Title/Abstract]) AND (autophag*[Title/Abstract] OR	Database inception to 15 July 2026; updated to 22 August 2026	121

lysosom*[Title/Abstract] OR mitophag*[Title/Abstract] OR ferritinophag*[Title/Abstract] OR lipophag*[Title/Abstract] OR ferroptos*[Title/Abstract] OR "lipid peroxidation"[Title/Abstract] OR "phospholipid peroxidation"[Title/Abstract] OR "iron homeostasis"[Title/Abstract] OR GPX4[Title/Abstract] OR SLC7A11[Title/Abstract] OR ACSL4[Title/Abstract])

PubMed search strategy used to support the focused narrative review. The literature search was initially completed on 15 July 2026, and the five search blocks were updated on 22 August 2026. PubMed was searched from database inception to the update date. No date, language, or publication-type filters were applied. The five search blocks returned 470 records before deduplication and 359 unique records after the results were combined by PMID.
